# The QOSMOS Study: Pharmacist-Led Multicentered Observational Study on Quality of Life in Multiple Sclerosis

**DOI:** 10.3390/neurolint13040065

**Published:** 2021-12-03

**Authors:** Vera Damuzzo, Laura Agnoletto, Roberta Rampazzo, Francesca Cammalleri, Luca Cancanelli, Marco Chiumente, Stefano Costantino, Silvia Michielan, Federica Milani, Alessia Sartori, Melania Rivano, Daniele Mengato

**Affiliations:** 1 Department of Pharmaceutical and Pharmacological Sciences, School of Hospital Pharmacy, University of Padua, 35122 Padua, Italy; silviamik89@gmail.com (S.M.); federicamilani90@gmail.com (F.M.); 2 National Association of Hospital Pharmacy Students-ReNaSFO, 20122 Milan, Italy; 3 Pharmacy, Santa Maria della Misericordia Hospital, 45100 Rovigo, Italy; laura.agnoletto@gmail.com; 4 Regional Pharmaceutical Office, Veneto Region, 30125 Venice, Italy; roberta.rampazzo67@gmail.com; 5 Department of Pharmaceutical Sciences, School of Hospital Pharmacy, University of Milan, 20122 Milan, Italy; cammallerifrancesca@gmail.com (F.C.); luca.cancanelli@gmail.com (L.C.); melania.rivano@gmail.com (M.R.); 6 Italian Society of Clinical Pharmacy and Therapeutics-SIFaCT, 20159 Milan, Italy; marco.chiumente@sifact.it (M.C.); daniele.mengato@gmail.com (D.M.); 7 Department of Drug Science and Technology, School of Hospital Pharmacy, University of Turin, 10124 Turin, Italy; stefano.costantino@aslcittaditorino.it; 8 Department of Food and Drug, School of Hospital Pharmacy, University of Parma, 43121 Parma, Italy; a.sartori2@ausl.pc.it; 9 Hospital Pharmacy Department, Azienda Ospedale-Università of Padova, 35121 Padua, Italy

**Keywords:** multiple sclerosis, quality of life, MS-QoL54, disease-modifying drugs, hospital pharmacist

## Abstract

Health-related quality of life is frequently included in patient-reported outcomes aimed at evaluating the effectiveness of disease-modifying drugs for multiple sclerosis, but recent data about Italian patients are missing. A multicenter observational and cross-sectional study was performed by students of hospital pharmacy to update existing data on quality of life and to correlate it with the pharmacological and medical history of patients. Quality of life (QoL) was assessed using the MS-QoL54 questionnaire, and the pharmacist collected patients’ characteristics, medical and pharmacological history, and Expanded Disability Status Scale (EDSS). Three hundred and forty-nine patients with multiple sclerosis were recruited from 16 centers between May 2018 and June 2019 (median age = 44.1 years; 68.9% women). The composite indexes of physical and mental well-being showed direct correlation with each other (R = 0.826; *p* < 0.001), and EDSS disability was an independent negative predictor of both indexes (R^2^ = 35.08% *p* < 0.001 and R^2^ = 15.74% *p* < 0.001, respectively). A trend of association between Physical Health Composite Score and different classes of oral disease-modifying drugs (DMDs) was observed. Our study found a decrease in QoL correlated with teriflunomide, which deserves further investigation. This experience demonstrates that joint action between scientific society and students association can be successful in conducting a no-profit multicenter observational study in a real-world setting.

## 1. Introduction

Multiple sclerosis (MS) is a chronic inflammatory autoimmune demyelinating disease of the central nervous system. Microglia, the brain resident immune cells, are highly activated and participate in all phases of the disease [1]. It is the second cause of disability in young adults after car accidents [2]. According to the Atlas of Multiple Sclerosis, edited by the Multiple Sclerosis International Federation and the World Health Organization, the disease affects 2.8 million people worldwide, with a prevalence of 36 per 100,000 people. Europe represents the region with the highest prevalence rate (133 per 100,000 people) in the world. Today there are more than 120,000 people in Italy suffering from MS, with a prevalence of more than 200 cases per 100,000 inhabitants [3]. Since 1980, many epidemiological studies have classified Italy as a high-risk area for MS, with the highest rates in the island of Sardinia and no evidence of a latitude gradient [4].

Despite pharmacological innovation in recent years, when disease progression occurs, there is an exacerbation of symptoms, so that monitoring patients’ disability is important to preserve their quality of life (QoL). The Expanded Disability Status Scale (EDSS) was designed and validated to measure the specific degree of disability in MS patients [5]. Higher EDSS scores, indicative of severe disability, are associated with lower QoL [6].

In addition to a clinical measure of disability provided by the EDSS, it is important to provide a measure of the QoL perceived by the patient since the disease onset. To this end, generic tools such as the 36-Item Short Form Survey (SF-36) can be used, but in the last decades, several MS-specific tools for QoL evaluation were developed [7]. The Multiple Sclerosis Quality of Life-54 (MSQOL-54) questionnaire was developed to combine the generic quality of life aspects from the SF-36 with 18 items exploring the issues related to physical disability and mental health most impacted by MS [8]. The questionnaire is available in 54 different languages, including Italian; each version has been validated and approved before being made available to clinicians [9]. Several studies evaluated the association between EDSS score, fatigue, dysarthria, the severity of symptoms, urinary incontinence, depression and anxiety, and QoL in MS patients using the MSQoL-54 instrument or other generic tools [10,11,12,13,14].

Beyond correlation between QoL and disease severity, disease-modifying drugs (DMDs) were demonstrated to ameliorate QoL of life in MS patients; there is a growing interest in investigating the impact of each DMD on QoL [15]. A recent review identified 37 studies in which the impact of DMDs on QoL was assessed, including 17 observational and 20 randomized clinical trials (RCTs) [16]. The tools used to measure QoL were generic tools in 12 cases (SF-36, SIP, SF-12), while specific tools for MS—or combinations of the two—were used in the remaining cases (MSQoL-54, MSIS-29, MSQLI, LMSQoL, FAMS, MusiQol, HAQUAMS, PRIMUS). IFN-β is the drug for which the largest number of studies is available, also due to the longer time elapsed since the start of its clinical use. IFN-β has been associated with better QoL in observational studies on relapsing–remitting multiple sclerosis, while this correlation is more evident in secondary progressive multiple sclerosis when only RCTs are considered.

Other studies on DMDs, as glatiramer acetate, teriflunomide or fingolimod, showed a modest impact on patients’ QoL, even though conflicting results have also been reported [16,17]. Dimethyl fumarate is one of the drugs for which QoL outcomes were studied more thoroughly, even in comparison with other drugs. In the two large pivotal trials, DEFINE and CONFIRM, the mean change in QoL in response to dimethyl fumarate was greater than in both the placebo and the glatiramer groups [18]. In a recent observational study, superiority was observed over glatiramer and IFN-β [19].

Our study aims to update the existing information on the QoL of Italian MS patients. This information mainly results from studies either monocentric or focused on only one treatment [14,20,21], and to estimate, in a real-world setting, the impact of disability and treatment on QoL of patients. The design of the study, which originated from joint action between a scientific society and a student association in the field of hospital pharmacy, focused our analysis on patients treated with self-administrable drugs and likely recruited patients with lower disability and higher QoL expectations.

## 2. Materials and Methods

### 2.1. Study Setting

Joint action was established between the National Association of Hospital Pharmacy Students (ReNaSFO) and the Italian Society of Clinical Pharmacy and Therapeutics (SIFaCT). Hospital pharmacy students were actively involved in the conduction of the study.

### 2.2. Study Design

QOSMOS was a multicentre, cross-sectional, non-interventional, observational trial to evaluate QoL in patients with MS in Italian clinical practice. The study was performed in collaboration with 16 Italian centers involved in the management and care of MS. The study lasted 12 months; patient enrolment was performed between May 2018 and June 2019.

The primary endpoint was to measure QoL in patients with MS through the MSQoL-54 questionnaire [9].

The secondary endpoints were: a correlation between MS-QoL54 scores and patients characteristics (age, sex); a correlation between MS-QoL54 scores and disease characteristics (type of MS, time from diagnosis, number of relapses in the previous two years, grade of disability measured by the Expanded Disability Status Scale (EDSS); a comparison of QoL data in relation to the pharmacological history of patients (number of drugs received from diagnosis, type of drug currently used, route of administration); recording patient satisfaction about the pharmaceutical assistance received.

### 2.3. Patient Characteristics

The inclusion criteria for eligible patients were:

Age ≥ 18 years;

MS diagnosis;

Treated by a self-administrated drug in any line of treatment.

Patients who needed to receive intravenous infusions of a medication to treat MS, aged <18 years or unable to provide informed consent were excluded. This study was conducted according to the Declaration of Helsinki and met Good Clinical Practice guidelines. The protocol was approved by central and local Ethics Committees and each site’s institutional review board. Patients included in this study provided informed consent, and patient clinical records were de-identified and analyzed anonymously.

### 2.4. Data Collection 

Medical and pharmacological anamnesis was obtained from the patient’s medical records. The data collected included: age, sex, type of MS, time from diagnosis, number of relapses in the last two years, EDSS score, drug history (drugs taken from diagnosis to time of observation) and current pharmacological treatment. During enrolment or through subsequent contacts, the patients received the validated MSQoL-54 questionnaire, consisting of 54 questions related to physical and mental health. The patient could choose to self-fill the survey, even in an online format, or to have it administered by healthcare professionals.

The MSQoL54 score is the result of a weighted combination of subscales and includes two main composite scores related to physical and mental health. QoL related to specific aspects can be evaluated considering the 12 subscales (physical function, role limitations-physical, role limitations-emotional, pain, emotional well-being, energy, health perceptions, social function, cognitive function, health distress, overall quality of life, and sexual function) and two single-item measures (satisfaction with sexual function and change in health).

All information collected, either directly from the patient or from the medical record, was recorded in an online case report form. All the patients were identified with a code to protect their identity. The case report form contains a section designed to record patients’ satisfaction with the drug distribution service and consulting by pharmacists, with the aim of detecting critical issues and suggestions for improving the pharmaceutical assistance of participant centers.

### 2.5. Statistical Analysis

Normally distributed continuous data were reported as the mean ± standard deviation and compared using the two-sided Student’s *t*-test. Non-normally distributed continuous data were reported as the median, interquartile-range (IQR), minimum and maximum values and compared using the Mann–Whitney test. As the number of patients in our study was less than 3000, normal distribution was studied using the Shapiro–Wilk test. A formal calculation of the sample size was not carried out due to the observational nature of the study and its descriptive approach. A Spearman correlation test was used to test the association between MSQoL-54 composite scores and EDSS score, the number of treatments previously received or the number of relapses in the last two years. A comparison between the MSQoL-54 Physical and Mental Health Scores of patients receiving different types of drugs was performed using ANOVA test with Tukey test for multiple comparisons. Univariate regression analysis was carried out considering MSQoL-54 scores as dependent variable and age, EDSS score, number of prior lines of therapy, number of relapses in the last two years and type of drug currently used as independent variables. All statistically significant parameters in the univariate analysis were introduced in a multivariate model to assess, which were independent predictors of QoL. The results were considered statistically significant for *p* < 0.05. No imputations for missing data were performed. Statistical analysis was performed using Minitab 17 Statistical Software.

## 3. Results

Three hundred and forty-nine patients out of the 320 required by the protocol were enrolled, with an average of 20 patients per center. Table 1 shows patients’ demographics, clinical characteristics and pharmacological history (Table 1).

Most of the patients were women (69%) and with a diagnosis of relapsing–remitting multiple sclerosis (93.5%). On average, patients were diagnosed multiple sclerosis seven years earlier (min = 0, max = 34 years) and had an EDSS score of 1.5 (min = 0, max = 7). The number of relapses in the last two years ranged from 0 to 6. Table 2 shows the distribution of EDSS scores according to different DMDs.

**Figure 1:**
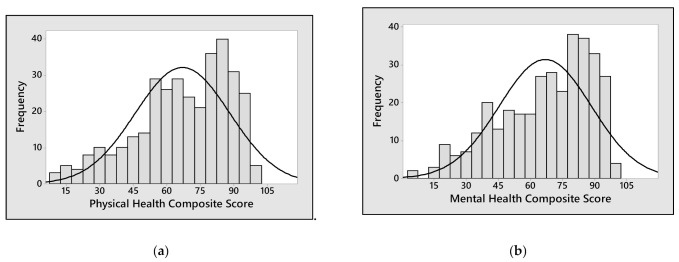

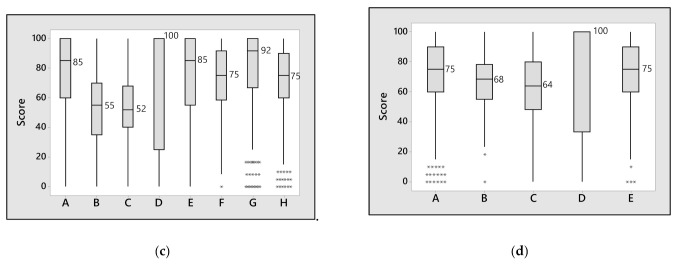
Overall QoL in MS patients. (**a**) Figure A shows the distribution of Physical Health Composite Score. (**b**) Figure b shows Mental Health Composite Score. (**c**) Figure C reports in box plots the subscales which contributes to Physical Health Composite Score calculation: A = Physical function, B = Health perceptions, C = Energy/fatigue, D = Role limitations–physical, E = Pain, F = Sexual function, G = Social function, H = Health distress. (**d**) Figure D illustrates the subscales composing the Mental Health Composite Score: A = Health distress, B = Overall quality of life, C = Emotional well-being, D = Role limitations–emotional, E = Cognitive function. Data falling outside the 1st and 3rd quartiles’ range are plotted as outliers of the data (*).

The most used pharmacological treatments were oral (57%). Dimethyl fumarate was used three times more frequently than teriflunomide and fingolimod. As for subcutaneous injection treatments, glatiramer acetate and interferon β-1a were used at a similar rate, while pegylated interferon was used less frequently. A minority of patients received a combined oral and subcutaneous drug regimen (1.7%) (Figure 1).

The most used first-line treatments were dimethyl fumarate (47/135 patients), interferon β-1a (38/135 patients), glatiramer acetate (20/135 patients) and teriflunomide (12/135 patients). Dimethyl fumarate was also the most prescribed second-line treatment (46/129 patients), followed by glatiramer acetate (27/129 patients), interferon β-1a (17/129 patients) and fingolimod (16/129 patients). 

The distribution of the two MSQoL-54 summary scores related to physical and mental health was shifted to the right towards 70 points, indicating an acceptable QoL (Figure 1a,b). A deeper insight into MSQoL-54 subscales showed that patients considered their QoL affected mainly by a low health perception and by fatigue, while sexual dysfunction and role limitations due to physical and emotional problems had a lower impact (Figure 1c,d).

**Figure 2:**
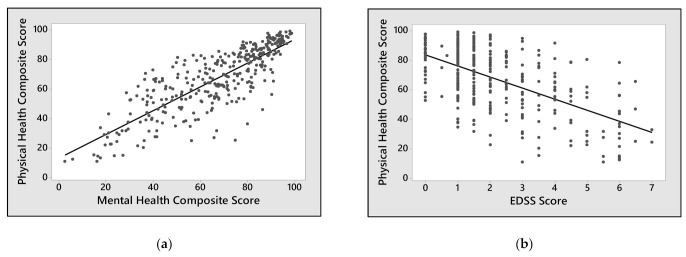

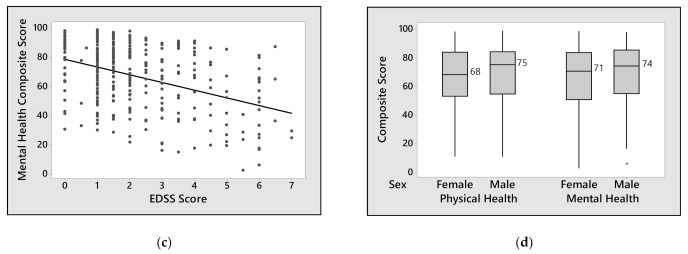
Correlation between MSQoL-54 Composite Scores and clinical characteristics of patients: (**a**) shows the correlation between Physical and Mental Health Composite Scores. (**b**,**c**) represent the correlation between EDSS Score and Physical or Mental Health Composite Scores, respectively. (**d**) shows the distribution of Physical or Mental Health Composite Scores among female and male participants. Data falling outside the 1st and 3rd quartiles’ range are plotted as outliers of the data (*).

The MSQoL-54 Physical and Mental Health Composite Scores directly correlated with each other (R = 0.826, *p* < 0.001) (Figure 2a) and they were both associated with patient’s degree of disability, expressed as EDSS score, according to an inverse relation (R = 0.511; *p* < 0.001 for Physical Health Composite Score (Figure 2b); R = 0.344; *p* < 0.001 for Mental Health Composite Score (Figure 2c)). There was no significant difference in terms of QoL between men and women, although there was a trend for better physical health-related QoL for men (Figure 2d). As expected, the EDSS score was found to be worse in patients with an increased number of prior lines of therapy (R = 0.176; *p* = 0.002) or with a higher number of relapses in the last two years (R = 0.172; *p* = 0.002). 

**Figure 3:**
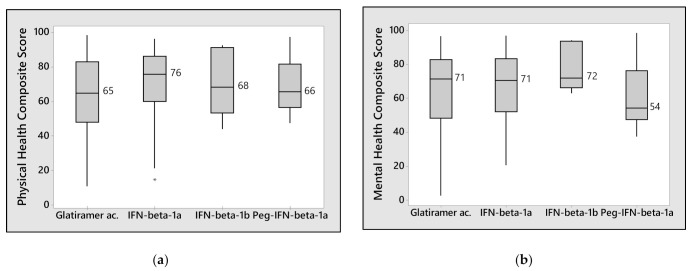

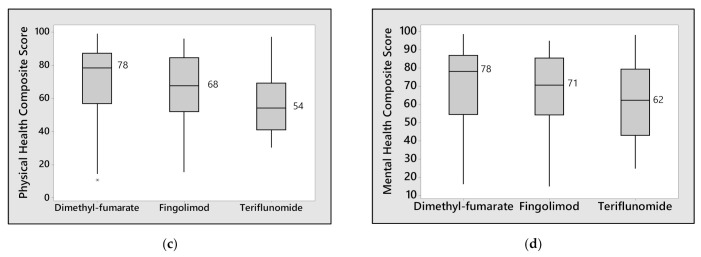
Association between MSQoL-54 Composite Scores and pharmacologic treatments: (**a**,**b**) illustrate the distribution of Physical and Mental Health Composite Scores in patients receiving drugs administrated through subcutaneous injection. (**c**,**d**) show the distribution of Physical and Mental Health Composite Scores in patients treated with oral drugs. Data falling outside the 1st and 3rd quartiles’ range are plotted as outliers of the data (*).

There was no difference in terms of QoL between patients taking DMD by oral route or by subcutaneous injection. In the subgroup analysis of drugs with the same route of administration, no major difference in QoL was found among patients receiving different treatments administered by subcutaneous injection (Figure 3a,b). Patients receiving subcutaneous peginterferon β-1a tended to have a lower psychological/mental well-being score than those treated with other subcutaneous drugs. Among patients taking oral drugs, there was a statistically significant reduction in the Physical Health Composite Score (*p* = 0.002; Figure 3c), and a trend in the Mental Health Composite Score of patients who were taking teriflunomide compared to those taking dimethyl fumarate (Figure 3d). 

**Figure 4:**
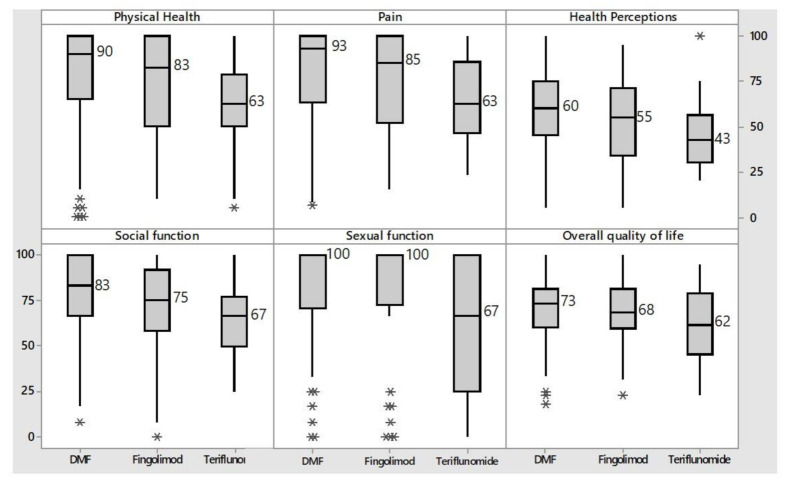
Distribution of the main subscales of MSQoL-54 influenced by oral drugs. DMF = dimethyl fumarate. Data falling outside the 1st and 3rd quartiles’ range are plotted as outliers of the data (*).

The QoL related to domains of physical function, pain, perception of one’s health and social and sexual functions was significantly lower in patients taking teriflunomide than in those treated with dimethyl fumarate (Figure 4).

A univariate linear regression analysis showed that the Physical Health Composite Score was negatively affected by age (R^2^ = 6.67%, *p* < 0.001), EDSS score (R^2^ = 33.77%, *p* < 0.001), number of relapses in the last two years (R^2^ = 4.45%, *p* < 0.001) and number of prior lines of therapy (R^2^ = 1.86%, *p* = 0.011) and from being currently treated with teriflunomide (R^2^ = 2.68%, *p* = 0.002). Dimethyl fumarate therapy was predictive of a better QoL (R^2^ = 1.67%, *p* = 0.016). There was no significant correlation with the other DMDs. In a subsequent multivariate analysis, the inverse relationship between Physical Health Composite Score and EDSS score or the intake of teriflunomide remained statistically significant (R^2^ = 35.08% *p* < 0.001). The results of univariate and multivariate regression of Physical Health Composite Score are presented in Table 3. 

In a similar linear regression model, the Mental Health Composite Score was negatively affected by the patient’s age (R^2^ = 1.32%, *p* = 0.032) and EDSS score (R^2^ = 16.28%, *p* < 0.001) number of relapses in the last two years (R^2^ = 3.37%, *p* = 0.001), and it tended to improve in patients taking dimethyl fumarate (R^2^ = 1.20%, *p* = 0.041). However, when the variables were compared through a multivariate model, only the negative correlation with the EDSS score remained statistically significant (R^2^ = 15.74% *p* < 0.001). The results of univariate and multivariate regression of the Mental Health Composite Score are presented in Table 4. 

The survey on patient satisfaction with the drug distribution service revealed that in two-thirds of cases, the drug was distributed in the hospital pharmacy by a pharmacist, while about 25% of patients preferred distribution in the ward by a nurse. Regardless of the place of drug delivery, 80% of participants referred to be satisfied with the distribution service and received detailed and comprehensible information on how to take the drug, its mechanism of action and its possible side effects. The negative aspects that emerged included difficulties in accessing the drug dispensing counter due to both short opening hours and physical obstacles (lack of parking, distance from the neurology department) and the supply of a single package of medication at a time.

## 4. Discussion

Our study showed an acceptable QoL in this series of patients, this finding being consistent with the low or intermediate degree of disability that characterized this cohort. Indeed, as in prior studies, we reported that QoL could be strongly affected by several factors such as age or disability status [22,23]. Our data confirmed the well-known inverse correlation between EDSS and QoL [6,14,24,25]. Of note, our results also indicated that the correlation involving physical and mental health scores was even stronger than that previously reported between EDSS and QoL.

One of the secondary endpoints of the study was to evaluate the impact of different DMDs on QoL. In fact, over the last decades, the development of this class of drugs has been focused not only on drug efficacy but also on preserving patients’ QoL. The direct consequence is that improvements in QoL are increasingly being included as endpoints in pivotal RCTs of the most recently approved DMDs, whereas for older drugs, such as interferon, QoL was mainly studied in post-marketing observational studies [17].

The results of the QOSMOS study in terms of correlation between DMDs and QoL are consistent with other real-world studies published in the literature [14,15]. Our results demonstrate that QoL is not dependent on the route of administration of the therapy. No trials are available from the literature comparing oral and subcutaneous therapies with QoL as an endpoint, but there are occasional comparisons between different molecules but with no evidence of the superiority of either route of administration. A 2014 open-label study, for example, observed QoL improvement in patients switched to fingolimod from subcutaneous IFN-β-1a or IFN-β-1b, but not from subcutaneous glatiramer or intramuscular IFN-β-1a [26].

Our study found that teriflunomide is an independent negative predictor of worse Physical Health Composite Score. In the literature, there are contrasting results on the association between teriflunomide and QoL: the follow-up of a phase II RCT on a small number of patients showed a slight decrease in QoL after 7 years of treatment with this drug, while a slight increase in QoL upon switching from other DMDs to teriflunomide was observed in an observational study [17,27]. We find this result interesting as teriflunomide was the DMD with the higher increase in prescriptions during 2017–2018 in Italy according to the Annual Report of Drug Prescription (OsMed) carried out by the Italian National Drug Agency [28,29]. 

On the other hand, our study demonstrated that dimethyl fumarate was associated with an improvement in several subscales of MSQoL-54, compared to teriflunomide, and was a predictor of better Physical Health Composite Score in a univariate regression analysis, although this result was not clearly confirmed by the multivariate regression model. The association between dimethyl fumarate and better QoL was often also reported in the context of RCTs such as DEFINE and CONFIRM studies, as well as in a combined analysis of the two trials [18,30,31]. Within these studies, a better QoL was observed for dimethyl fumarate compared to placebo and glatiramer. The superiority of dimethyl fumarate over glatiramer and IFN-β, in terms of QoL, was also observed in a recent observational study [19].

Finally, among the patients taking subcutaneous drugs, those treated with peginterferon β-1a tended to have a lower psychological/mental health score than those treated with other drugs based on the same route of administration. This finding is an original result since there are no direct comparisons in terms of QoL between these specific drugs. However, this finding is not in line with the 2018 RCT by Hendin B et al., in which patients expressed greater satisfaction after switching from IFN-β to PEG-IFN-β [32]. It should be stressed that the authors did not use an overall QoL assessment but a specific test on satisfaction about drug therapy (TSQM).

Our study has several limitations. Firstly, the lack of randomization or propensity-matched controls does not allow us to draw definite conclusions on the association between QoL and DMDs. Moreover, the cross-sectional design of the study hampers to track the possible changes in QoL from baseline through follow-up. Albeit preliminary, our results highlight the need for further real-life studies in which the different available pharmacological regimens are included in a multivariate model along with other impacting factors such as EDSS, age and cognitive performance. 

As it is a multicenter study, our results about the satisfaction with drug distribution procedures were strongly influenced by local differences between different hospitals, and they must therefore be interpreted as descriptive data. The trend shows a generally high level of patient satisfaction regardless of the context in which the distribution of drugs takes place, an index of a good efficiency of the different health care services. Participating in the study was an opportunity for the local centers to analyze some critical issues in their organizational models and evaluate possible improvements (e.g., more flexible dates and times compatibly with available resources, home deliveries for the most fragile patients).

## 5. Conclusions

Our data confirmed the inverse correlation between EDSS and QoL and the linear correlation between QoL related to physical and mental health. As it regards the impact of different DMDs on QoL, our study found that teriflunomide is an independent negative predictor of worse Physical Health Composite Score and that dimethyl fumarate is associated with an improvement in several subscales of MSQoL-54. Another finding is that patients treated with peginterferon β-1a tended to have a lower psychological/mental health score than those treated with other drugs with the same route of administration. Regarding the pharmaceutical assistance received by patients, our multicenter study demonstrated a high level of patient satisfaction with drug distribution services and consulting by pharmacists.

The joint action of a scientific society (SIFaCT—Italian society for clinical pharmacy and therapeutics) and an association of postgraduate students (National Association of Hospital Pharmacy Students, ReNaSFO) is an unprecedented method to conduct observational studies in real practice. This method achieved two main goals: on the one hand, it allowed hospital pharmacy students to gain specific experience in clinical research, in line with the recommendation by the national program of specialization schools. On the other, the widespread distribution of specialization schools throughout the country facilitated the recruitment of many participating centers representative of different regions. This synergy has led to achieving the main study aims and can hopefully be replicated for other real-life studies in various clinical fields, given the transversal role of hospital pharmacists within our National Health Care System [33].

## Figures and Tables

**Table 1:** Clinical characteristics of patients.

Number of Enrolled Patients		349
Sex	Female [*n* (%)]	241 (69)
	Male [*n* (%)]	108 (31)
Age [median (min, max)]		44 (0, 75)
Disease [*n* (%)]	Relapsing–Remitting MS (RRMS)	325 (93.1)
	Secondary-Progressive MS (SPMS)	12 (3.4)
	Progressive-Relapsing MS (PRMS)	3 (0.9)
	Primary-Progressive MS (PPMS)	1 (0.3)
	Other (Clinically isolated syndrome, partial transverse myelitis)	8 (2.3)
EDSS Score at enrolment [median (min, max)]		1.5 (0.0, 7.0)
Number of relapses in previous two years [median (min, max)]		0.0 (0.0, 6.0)
Time since diagnosis-years [median (min, max)]		6.8 (0, 34.4)
Current treatment [*n* (%)]	Azathioprine	2 (0.6)
	Dimethyl fumarate	121 (34.7)
	Fingolimod	42 (12.0)
	Glatiramer acetate	59 (16.9)
	Interferon β-1a	61 (17.5)
	Interferon β-1b	5 (1.43)
	Peg-Interferon β-1a	19 (5.44)
	Teriflunomide	34 (9.7)
	Combined regimens	6 (1.7)
Number of drugs used previously [median (min, max)]		1 (0.0, 4.0)

**Table 2:** Distribution of EDSS score’s values according to the type of DMD.

Treatment	*n*	Missing	Minimum	Q1	Median	Q3	Maximum
Dimethyl fumarate	114	7	0	1	1.5	3	7
Interferon β-1a	58	3	0	1	1.5	2.125	6
Glatiramer acetate	53	6	0	1	2.5	4	6.5
Fingolimod	40	2	0	1.125	2	3.875	6.5
Teriflunomide	32	2	0	1	2	3.875	6
Peg-Interferon β-1a	16	3	0	1	1.5	2	3
Combined regimens	5	0	1	1	1	1.5	2
Interferon β-1b	4	1	1	1	1.25	4.88	6
Azathioprine	2	1	5	*	5.5	*	6

1st and 3rd quartiles (Q1–Q3) can not be calculated for azathioprine group due to insufficient sample number (*n* = 2). Data falling outside the 1st and 3rd quartiles’ range are plotted as outliers of the data (*).

**Table 3:** Univariate and multivariate linear regression analysis: standardized Beta coefficients of independent variables and *p*-values assessed for Physical Health Composite Score.

	Univariate Analysis		Multivariate Analysis	
	Standardized β	*p*-Value	Standardized β	*p*-Value
Age	−0.4632	<0.001	0.0038	0.968
n° of relapses in the last two years	−4.94	<0.001	−2.26	0.039
n° of prior treatments	−3.02	0.011	−0.75	0.468
EDSS score	−7.509	<0.001	−7.207	<0.001
Dimethyl fumarate	5.72	0.016	2.17	0.294
Interferon β-1a	4.21	0.156		
Glatiramer acetate	−5.01	0.096		
Fingolimod	−0.94	0.787		
Teriflunomide	−11.62	0.002	−9.7	0.003
Peg-Interferon β-1a	2.17	0.664		
Combined regimens	2.26	0.812		
Interferon β-1b	4.33	0.649		

**Table 4:** Univariate and multivariate linear regression analysis: standardized Beta coefficients of independent variables and *p*-values assessed for Mental Health Composite Score.

	Univariate Analysis		Multivariate Analysis	
	Standardized β	*p*-Value	Standardized β	*p*-Value
Age	−0.21	0.032	0.142	0.198
n° of relapses in the last two years	−4.4	0.001	−2.48	0.055
n° of prior treatments	−1.11	0.361		
EDSS score	−5.339	<0.0001	−5.309	<0.001
Dimethyl fumarate	4.96	0.041	3.28	0.165
Interferon β-1a	0.85	0.780		
Glatiramer acetate	−2.52	0.413		
Fingolimod	1.06	0.766		
Teriflunomide	−5.94	0.127		
Peg-Interferon β-1a	−5.43	0.286		
Combined regimens	−3.84	0.693		
Interferon β-1b	11.19	0.249		

## Data Availability

The MSQoL-54 and clinical data used to support the findings of this study are restricted according to the Ethics Boards of participating centers in order to protect patient privacy. Data are available, upon request, from the Italian Society of Clinical Pharmacy and Therapeutics (segreteria@sifact.it) for researchers who meet the criteria for access to confidential data.
